# Fast and sensitive differential diagnosis of pseudorabies virus-infected versus pseudorabies virus-vaccinated swine using CRISPR-Cas12a

**DOI:** 10.1128/spectrum.02617-23

**Published:** 2023-12-11

**Authors:** Hao Wang, Hongzhao Li, Bo Tang, Chen Ye, Meiqing Han, Lin Teng, Min Yue, Yan Li

**Affiliations:** 1 Department of Veterinary Medicine, Institute of Preventive Veterinary Sciences, Zhejiang University College of Animal Sciences, Hangzhou, Zhejiang, China; 2 Hainan Institute of Zhejiang University, Sanya, Hainan, China; 3 Institute of Veterinary Immunology and Engineering, Jiangsu Academy of Agricultural Sciences, Nanjing, Jiangsu, China; 4 Zhejiang Provincial Key Laboratory of Preventive Veterinary Medicine, Hangzhou, Zhejiang, China; 5 State Key Laboratory for Diagnosis and Treatment of Infectious Diseases, National Clinical Research Center for Infectious Diseases, National Medical Center for Infectious Diseases, The First Affiliated Hospital, Zhejiang University School of Medicine, Hangzhou, Zhejiang, China; University of Prince Edward Island, Charlottetown, Prince Edward Island, Canada

**Keywords:** pseudorabies virus, PRV, diagnostic, CRISPR-Cas12a, nucleic acid detection

## Abstract

Pseudorabies virus (PRV) is the causative pathogen of Aujeszky’s disease. It causes high mortality and miscarriage rates in the infected swine, leading to tremendous economic losses in the global swine industry. PRV has also been demonstrated to trigger viral encephalitis in humans. The eradication policy and large-scale vaccination have been adopted globally as the most effective strategy against PRV. A simple, fast, and sensitive diagnostic method is highly demanded to differentiate between vaccinated and infected swine. Herein, we designed a detection system combining multienzyme isothermal rapid amplification (MIRA) and CRISPR-Cas12a (termed MIRA-Cas12a), characterized by high sensitivity and specificity, low cost, less equipment, and convenient visualization. By targeting the gB, gE, and TK genes of PRV, the MIRA-Cas12a assay is able to distinguish the infected, uninfected, and vaccinated swine by the naked eyes in 40 min (from DNA extraction to result readout) and with comparable sensitivity to conventional quantitative PCR. A 37°C heater and a source of blue light are all the equipment required to detect PRV. Thus, the MIRA-Cas12a detection will facilitate PRV surveillance and minimize the financial losses to the swine industry.

**IMPORTANCE:**

Pseudorabies virus (PRV) causes high mortality and miscarriage rates in the infected swine, and the eradication policy coupled with large-scale vaccination of live attenuated vaccines has been adopted globally against PRV. Differential diagnosis of the vaccinated and infected swine is highly demanded. Our multienzyme isothermal rapid amplification (MIRA)-Cas12a detection method described in this study can diagnose PRV with a superior sensitivity comparable to the quantitative PCR (qPCR) and a competitive detection speed (only half the time as qPCR needs). The portable feature and the simple procedure of MIRA-Cas12a make it easier to deploy for clinical diagnosis, even in resource-limited settings. The MIRA-Cas12a method would provide immediate and accurate diagnostic information for policymakers to respond promptly.

## INTRODUCTION

Pseudorabies or Aujeszky’s disease (1) is a worldwide notifiable infectious disease for wild boar and domestic pigs of all ages. This highly contagious swine disease is caused by the pseudorabies virus (PRV). Once infected, it largely reduces the production performance of the adult and triggers 100% mortality of piglets. Many other mammals are also susceptible to PRV or can become carriers (2). Nationwide vaccination with gE- or/and TK-deleted vaccines of PRV has long been widely implemented in farms to prevent the spread of PRV (3).

PRV is an enveloped virus, and its genome consists of double-stranded DNA (dsDNA) of ~150 kb, encoding at least 11 glycoproteins (gB, gC, gD, gE, gG, gH, gI, gK, gL, gM, and gN). These genes have been demonstrated to engage in replication, immunogenicity and pathogenicity (4). The viral glycoproteins gB and gH are essential for PRV replication, while gE and TK are not essential for viral replication but are crucial for viral invasion and transmission (5). As the primary virulent genes of PRV, gE and TK are often naturally or artificially deleted from the genome to create attenuated vaccine strains of PRV (3), such as Bartha-K61, HB-98, SA215, NY, HNX, and Bucharest strains (6 – 10). Bartha-K61 is the first naturally attenuated PRV strain developed by deleting gE in 1961 (11), which has been widely used for PRV prevention since its establishment (10, 12). Other vaccines of PRV with single or multiple deletions in genes, such as gI, US2, and US9, have also been reported (13). The concept of the differentiation of infected from vaccinated animals (DIVA) was brought by Jan T. van Oirschot in 1999 (14) and laid down the foundation of PRV eradication worldwide since then (3). With continuous large-scale vaccination with gene-deleted vaccines and a DIVA strategy, PRV has been eradicated from domestic pigs in several European countries (15). However, it is still a fatal epidemic disease widely prevalent in Asia (2), and a more efficient PRV diagnostic method for DIVA is in high demand.

Detection of anti-gE antibodies is the most widely used antibody-based method, such as the gE-enzyme-linked immunosorbent assay (16) and the lateral flow immunoassay (17), which is efficient and easy-to-operate for the detection. However, it might take several days to produce antibodies after PRV infection, during which the virus may already spread through the entire farm. The quantitative PCR (qPCR) method features superior sensitivity [mainly with a detection limit of ~×10^1^ copies/reaction (18 – 21)] and specificity. Still, the requirement of special equipment limits its application in the frontline against PRV, especially with resource-lacking settings. Thus, it remains necessary to establish a time- and cost-effective, simple, and accurate diagnostic method to differentiate the PRV-infected and immunized swine.

The clustered regularly interspaced short palindromic repeat (CRISPR) technology has emerged as a powerful next-generation diagnostics thanks to its extraordinary specificity, speediness, and convenience, such as DNA Endonuclease Targeted CRISPR Trans Reporter (DETECTR) and Specific High Sensitivity Enzymatic Reporter UnLOCKing (SHERLOCK) (22). In this study, we developed a fast, sensitive, and specific CRISPR-based method for the detection and differentiation of the wild-type PRV and gene-deleted vaccine strains by combining Cas12a with multienzyme isothermal rapid amplification (MIRA) (referred to as MIRA-Cas12a detection). MIRA is an isothermal DNA amplification method developed from recombinase polymerase amplification (23). By targeting gB (contained in both wild-type PRV and vaccine strains) and gE and TK (deleted in vaccine strains) of PRV, our method is able to identify the vaccinated and infected swine in 40 min with a high sensitivity comparable to qPCR. Furthermore, all the equipment needed for MIRA-Cas12a detection is a 37°C heater and a source of LED blue light. The CRISPR-based PRV detection will largely sharpen PRV monitoring and reduce the associated financial cost.

## MATERIALS AND METHODS

### Primers, crRNAs, and reporters

All the sequences of primers and reporters used in this study were synthesized by Tsingke Biotechnology Co., Ltd. (Nanjing, China) and listed in Table 1. The complete coding sequences of gB, gE, and TK genes of PRV were amplified from PRV DF strain (a wild-type PRV isolated from a brain of PRV-infected swine in Jiangsu Province, China) by PCR with 2× Phanta Master Mix (Vazyme Biotech, Nanjing, China) and then purified by EasyPure Quick Gel Extraction Kit (Easter Biology Co., Ltd., Zhejiang, China). The DNA fragments were inserted into the pIERS2-EGFP vector via EcoR I and BamH I (Vazyme Biotech, Nanjing, China). The gB, gE, and TK genes in the PRV DF strain were sequenced by Sanger sequencing, and these genes share identical sequences with those of the PRV DX strain (GenBank: MZ063026.1).

**TABLE 1:** A list of primers, crRNAs, and reporter used in this study^*a*^

Name	Sequence
gB-crRNA-1	UAAUUUCUACUAAGUGUAGAUUGCGCACGCCGCACUUCACG
gB-crRNA-2	UAAUUUCUACUAAGUGUAGAUCGCGCCUGCAGUUCACCUAC
gB-crRNA-3	UAAUUUCUACUAAGUGUAGAUAGCUGGGCGGCGGGUACGUG
gE-crRNA-1	UAAUUUCUACUAAGUGUAGAUUCCGGAUCGCGGAACCAGAC
gE-crRNA-2	UAAUUUCUACUAAGUGUAGAUUGGCGGUGGGCGACCGGCCG
gE-crRNA-3	UAAUUUCUACUAAGUGUAGAUUGCUGGCGCUGGGCUCCUUC
TK-crRNA-1	UAAUUUCUACUAAGUGUAGAUGGCGCGUACAAGGCGCCCGA
TK-crRNA-2	UAAUUUCUACUAAGUGUAGAUGGGCCCGCGGUCGAGGGCCC
TK-crRNA-3	UAAUUUCUACUAAGUGUAGAUACCGCCACCCGGUGGCCGCG
gB-sPAM-crRNA	UAAUUUCUACUAAGUGUAGAUUUGUAGCGCCGCCGGUAGAU
gE-sPAM-crRNA	UAAUUUCUACUAAGUGUAGAUUACACCAGCCUGCCCACGCA
MIRA-gB-F	CGTGTACATGTCCCCCTTCTAC
MIRA-gB-R	GGATCATCTCCTCGGCCT
MIRA-gE-F	CACATGCTCTCTCCGGTGT
MIRA-gE-R	TCGTCACTTCCGGTTTCTCC
MIRA-TK-F	AGCTCCAGGACACCCTCTT **T** CGG
MIRA-TK-R	ACACGCACTGCCGGATGTGG
sPAMC-gB-F	AGGCCGAGGAGATGATCC
sPAMC-gB-R	CCAGCTCGTTCGAGATCAG
sPAMC-gE-F	CACATGCTCTCTCCGG **T** TGT
sPAMC-gE-R	TCGTCACTTCCGGTTTCTCC
qPCR-F	CGCAACAACCACAAGGTGA
qPCR-R	TGGACAGGGCGAAGGAG
PCR-F	CGCAACAACCACAAGGTGA
PCR-R	TGGACAGGGCGAAGGAG
ssDNA reporter	FAM-TTATT-BHQ1

^*a*^ Note that the underlined letter indicates the inserted base in the primer for creating a PAM.

### Vaccines against swine viruses

The PRV vaccine strains used in this study included Bartha-k61 (Harbin Pharmaceutical Group Bio-vaccine Co., Ltd., Heilongjiang, China), C strain (Yangzhou Youbang Biological Medicine Co., Ltd., Jiangsu, China), and HB-98 strain (Wuhan Keqian Biology Co., Ltd., Hubei, China). The vaccines of other swine viruses included swine fever virus (CFV) vaccine (Guangdong Wen’s Dahua Nong Biotechnology Co., Ltd., Guangdong, China), the bivalent vaccine of porcine epidemic diarrhea virus (PEDV) (AJ1102-R strain) and transmissible gastroenteritis virus (TGEV) (WH-1R strain) (YEBIO, Shandong, China), and porcine reproductive and respiratory syndrome virus (PRRSV) vaccine (CH-1R strain) (Wuhan Keqian Biology Co., Ltd., Hubei, China). The genomic nucleic acid from vaccines and clinic samples of PRV were extracted by Easy Tissue & Blood DNA Extraction Kit (Easter Biology Co., Ltd., Zhejiang, China). The PRV vaccines Bartha-k61 and C are gE-deleted, and HB-98 is TK-deleted, while vaccines of other viruses possess intact genomes. All the vaccines we used in the study were the live-attenuated ones. The three clinical samples stored in our lab were collected from the brains of two sick sows with reproductive failure and neurological symptoms (Samples 1 and 2) and one healthy pig (Sample 3) on a farm in Jiangsu Province, China. The viral loads of Samples 1 and 2 are 3,162 copies/µL, detected by qPCR. The other 36 clinical samples were collected from healthy swine in a slaughterhouse in Zhejiang Province, China.

### The crRNA selection and preparation

To determine the most conserved regions as the target, we downloaded sequences of gB, gE, and TK genes from different PRVs from the NCBI database and aligned the sequences with the MEGA-X program. The CRISPR RNAs (crRNAs) were designed against the conserved regions using the online website (CHOPCHOP, https://chopchop.cbu.uib.no/), and the optimized crRNA was selected for Cas12a cleavage assay.

The crRNAs against target genes were generated by *in vitro* transcription assay with T7 RNA polymerase (Vazyme Biotech, Nanjing, China). A single-stranded DNA (ssDNA) containing T7 promotor and crRNA sequence (SUNYA Biotechnology Co., Ltd., Zhejiang, China) were annealed to its complementary oligonucleotides, and the produced dsDNA served as the template for T7 transcription. Briefly, 8 µL of template dsDNA (100 ng/µL), 8 µL of NTP buffer mix, 2 µL of T7 RNA polymerase mix, and 2 µL reaction buffer were mixed and incubated at 37°C for 16 h. The transcription product was purified by Monarch RNA Cleanup Kit (New England Biolabs, Massachusetts, USA). The concentration of the purified crRNA was measured using a NanoDrop 2000 (Thermo Fisher Scientific, Massachusetts, USA). All crRNAs were aliquoted and stored at −80°C after purification. The crRNAs targeting gB are gB-crRNA-1 (genome location: 18,455–18,474), gB-crRNA-2 (genome location: 17,927–17,946), and gB-crRNA-3 (genome location: 17,519–17,538). The crRNAs targeting TK are TK-crRNA-1 (genome location: 61,078–61,097), TK-crRNA-2 (genome location: 60,643–60,662), and TK-crRNA-3 (genome location: 60,686–60,705). The crRNAs targeting gE are gE-crRNA-1 (genome location: 126,166–126,185), gE-crRNA-2 (genome location: 125,132–125,151), and gE-crRNA-3 (genome location: 125,813–125,832). Sequences of the crRNA are present in Table 1

### Multienzyme isothermal rapid amplification

The amplification of target nucleic acid was performed using the MIRA Kit (Weifang AMP-Future Biotech Co., Ltd., Weifang, China). According to the manufacturer’s protocol, the reaction was prepared in a tube containing 29.4 µL of buffer A, 2 µL of forward primers (10 µM) of gB, gE, and TK gene, 2 µL of reverse primers (10 µM) of gB, gE, and TK gene, 5.1 µL of nuclease-free water, and 1 µL of dsDNA template. Two-microliter buffer B was finally added to the mixture. After incubating at a 37°C water bath for 15 min, 2 µL of the amplification product was transferred into Cas12a cleavage reaction. To avoid aerosol pollution, we added 20 µL mineral oil (Sigma-Aldrich, Massachusetts, USA) to the MIRA reaction before incubation at 37°C. The MIRA products of gB, gE, and TK are 248, 265, and 240 bp, respectively.

### Cas12a cleavage assay

Firstly, 1 µL LbCas12a protein (100 nM) (Bio-lifesci, Guangzhou, China) and 1 µL crRNA (140 nM) were mixed and incubated at 37°C for 5 min to preassemble the complex of LbCas12a-crRNA. Then, the mix was added with 1 µL ssDNA reporter (10 µM) (SUNYA Biotechnology, Zhejiang, China), 1 µL 10× NEBuffer 2.1 (New England Biolabs, Massachusetts, USA), 2 µL MIRA product, and 4 µL nuclease-free water. The reaction was incubated at 37°C for 10 min, and results were read out under an LED blue light transilluminator (THBC-470) (Tuohe Electromechanical Technology Co., Ltd., Shanghai, China).

Serial dilutions of plasmid containing gB (1.4, 2.8, and 5.6, 1.4 × 10^1^, 2.8 × 10^1^, 2.8 × 10^2^, 2.8 × 10^3^, and 2.8 × 10^4^ copies/μL), plasmid containing gE (3.3, 6.6, and 1.3 × 10^1^, 3.3 × 10^1^, 6.6 × 10^1^, 6.6 × 10^2^, 6.6 × 10^3^, and 6.6 × 10^4^ copies/μL), or plasmid containing TK (3.1, 6.3, and 1.2 × 10^1^, 3.1 × 10^1^, 6.3 × 10^1^, 6.3 × 10^2^, 6.3 × 10^3^, and 6.3 × 10^4^ copies/μL) were used as the template for MIRA to test the sensitivity of MIRA-Cas12a assay, respectively. After amplification, the products were pipetted into the corresponding Cas12a-crRNA mix. These reactions were incubated in a real-time qPCR system (Bio-Rad, Watford, UK) for about 10 min at 37°C, and the generated fluorescent signals were recorded every 30 s. Several vaccines of swine viruses, including PRRSV, CFV, PEDV, and TGEV, were used to analyze the speciﬁcity.

### Polymerase chain reaction

Tenfold serial plasmid diluents containing gB with a concentration ranging from 2.8 × 10^2^ to 2.8 × 10^7^ copies/μL were prepared as a template of PCR. The reaction included 10 µL 2× Phanta Master Mix, 0.5 µL forward primer, 0.5 µL reverse primer, 1 µL plasmid, and 8 µL nuclease-free water. PCR program was 95°C for 3 min, followed by 35 cycles of 95°C for 15 s, 57°C for 15 s, and 72°C for 10 s; the final extension was 72°C for 5 min. The PCR products were analyzed by gel electrophoresis.

### Quantitative PCR

Since qPCR has always been accepted as a reliable methodology for pathogen detection, we used it as a reference approach to evaluate our diagnostic method. The plasmid containing gB was 10-fold serially diluted to 2.8–2.8 × 10^7^ copies/μL, each used as the template for qPCR. The reaction consisted of 5 µL 2× Taq Pro Universal SYBR qPCR Master Mix (Vazyme Biotech, Nanjing, China), 0.25 µL forward primer (10 µM), 0.25 µL reverse primer (10 µM), 3.5 µL nuclease-free water, and 1 µL template DNA. The reaction procedure included heat activation at 95°C for 30 s, followed by 40 cycles of 95°C for 30 s, 60°C for 20 s, and 72°C for 10 s.

### The sPAMC assay

The suboptimal protospacer adjacent motif (sPAM) assay based on CRISPR/-Cas12a (sPAMC) was performed in 30 µL reaction volumes containing 33 nM LbCas12a-crRNA complex, 400 nM ssDNA FQ reporter, dsDNA substrate (10-fold dilution series of plasmids containing gB with a concentration ranging from 3.3 × 10^2^ to 3.3 × 10^6^ copies/μL or plasmid containing gE with a concentration ranging from 3.8 × 10^2^ to 3.8 × 10^6^) and MIRA components (24). The Cas12a-crRNA complex (2 µL), ssDNA FQ reporter (8 µL), and MIRA mixture (18 µL) were added to a one-pot reaction. Finally, 1 µL target dsDNA and 1 µL buffer B were pipetted into the reaction and incubated in a fluorescence quantitative instrument at 37°C for 15 min.

### Detection of cultured PRV diluents by qPCR and MIRA-Cas12a

For viral titration, 7 × 10^3^ PK-15 cells per well were plated in 96-well plates and cultured with Dulbecco’s modified Eagle’s medium (DMEM) containing 10% fetal bovine serum (FBS). When the cells grew to ~90% confluence, the medium was replaced with DMEM supplemented with 2% FBS, and the 10-fold serially diluted viral supernatants were added into the medium. The cytopathic effect was observed for 72–96 h. TCID_50_ of PRV on PK-15 was then determined using the Reed and Muench method (25). For detection of PRV nucleic acid, 200 µL PRV supernatant was 10-fold diluted and then processed by TIANamp Virus DNA Extraction Kit (TIANGEN Company, Beijing, China). DNA was eluted with 40 µL water. One-microliter extracted DNA of different dilutions was added to the reaction systems of qPCR and MIRA-Cas12a to measure their detection limit.

## RESULTS

### The schematic diagram of MIRA-Cas12a detection on differentiation of vaccine and wild type of PRV

**Fig 1:**
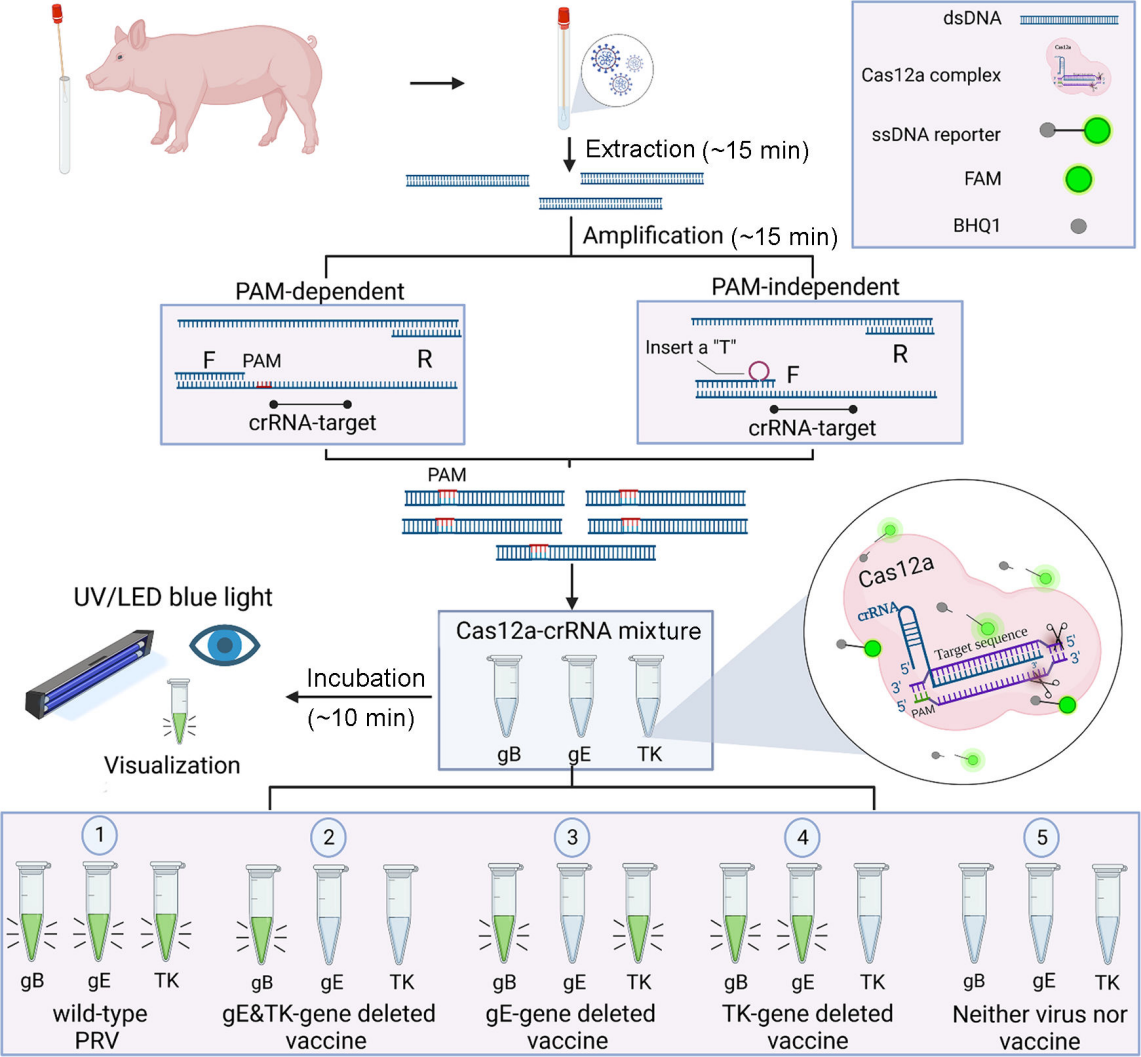
The schematic of MIRA-Cas12a-based differential diagnosis of wild-type PRV and the gene-deleted PRV vaccines. MIRA amplification was performed after the nucleic acid was extracted from the sample. For the targeted conserved sequence of gB or gE containing a PAM (“TTTN”) for Cas12a, the amplified product will include a natural PAM for following Cas12a cleavage, termed the PAM-dependent manner; for the targeted conserved sequence of TK gene without PAM, we inserted a “T” base behind “TT” at three nucleotides to the 3′ end of forward primer, so that the PAM sequence (“TTTN”) is artificially introduced into the amplicon through MIRA amplification. This method allows us to detect any target using CRISPR-Cas in a PAM-independent manner. Then, the amplification product was transferred to and incubated with Cas12a-crRNA complex targeting gB, gE, and TK for about 10 min. Five types of results will be observed: (1) the fluorescence signals in all three tubes indicate the swine infected with wild-type PRV; (2) the sole fluorescence signal in the tube detecting gB indicates the swine vaccinated with gE- and TK-deleted vaccines; (3 and 4) the fluorescence signals in the tube detecting gB and either gE or TK gene indicate the swine vaccinated with the gE- or TK-deleted vaccines; and (5) no fluorescence signal in all tubes means that the swine is neither infected with wild-type PRV nor vaccinated.

To detect and differentiate the vaccine and wild type of PRV, we have established a pipeline of MIRA-Cas12a detection (Fig. 1). As the gB gene exists in both PRV live-attenuated vaccines and wild-type PRV and gE or/and TK is deficient in vaccines (Table 2), the triple detection on gB, gE, and TK determines whether the swine harbored PRV vaccine or wild-type virus. After extracting the nucleic acid from the sample, we amplified targeted genes with MIRA. As the targeted conserved sequence of gB or gE with a PAM (“TTTN”), the amplified product contained a PAM for the following Cas12a recognition, termed the PAM-dependent manner. While the targeted conserved sequence of the TK gene does not include a PAM, we designed a primer adjacent to the target sequence against crRNA and created a PAM by inserting a T base behind “TT” in forward primer at three nucleotides to the 3′ end, which is termed as PAM-independent manner. The PAM sequence is incorporated in the amplicons by MIRA. After amplification, the products were transferred to the corresponding Cas12a/crRNA mix. Five different results will be read out under the LED blue light: (i) the fluorescence signals in all three tubes indicate the swine infected with wild-type PRV; (ii) the sole fluorescence signal in the tube detecting gB indicates the swine vaccinated with gE- and TK-deleted vaccines; (iii and iv) the fluorescence signals in the tube detecting gB and either gE or TK gene indicate the swine vaccinated with the gE- or TK-deleted vaccines; and (v) no fluorescence signal in all tubes means that the swine is neither infected with wild-type PRV nor vaccinated.

**TABLE 2:** A list of widely used live-attenuated PRV vaccines

Vaccine strain	Deleted gene(s)	Reference
Bartha-K61^*a*^	gE & gI	Bartha (11)
HB-98^*a*^	TK & gG	Fang et al. (26)
C strain^*a*^	gI & gE & Us9 & Us2	A naturally deficient PRV isolated from China
HB2000	TK & gE & gI	Wang et al. (27)
BUK	gE	Skoda et al. (28)
SA215	gE & gI & TK	Zhu et al. (29)
HNX	gE & TK	Yao et al. (9)
NY	gE & gI & TK	Zhao et al. (6)

^*a*^ Indicates the vaccine used in our study.

**Fig 2:**
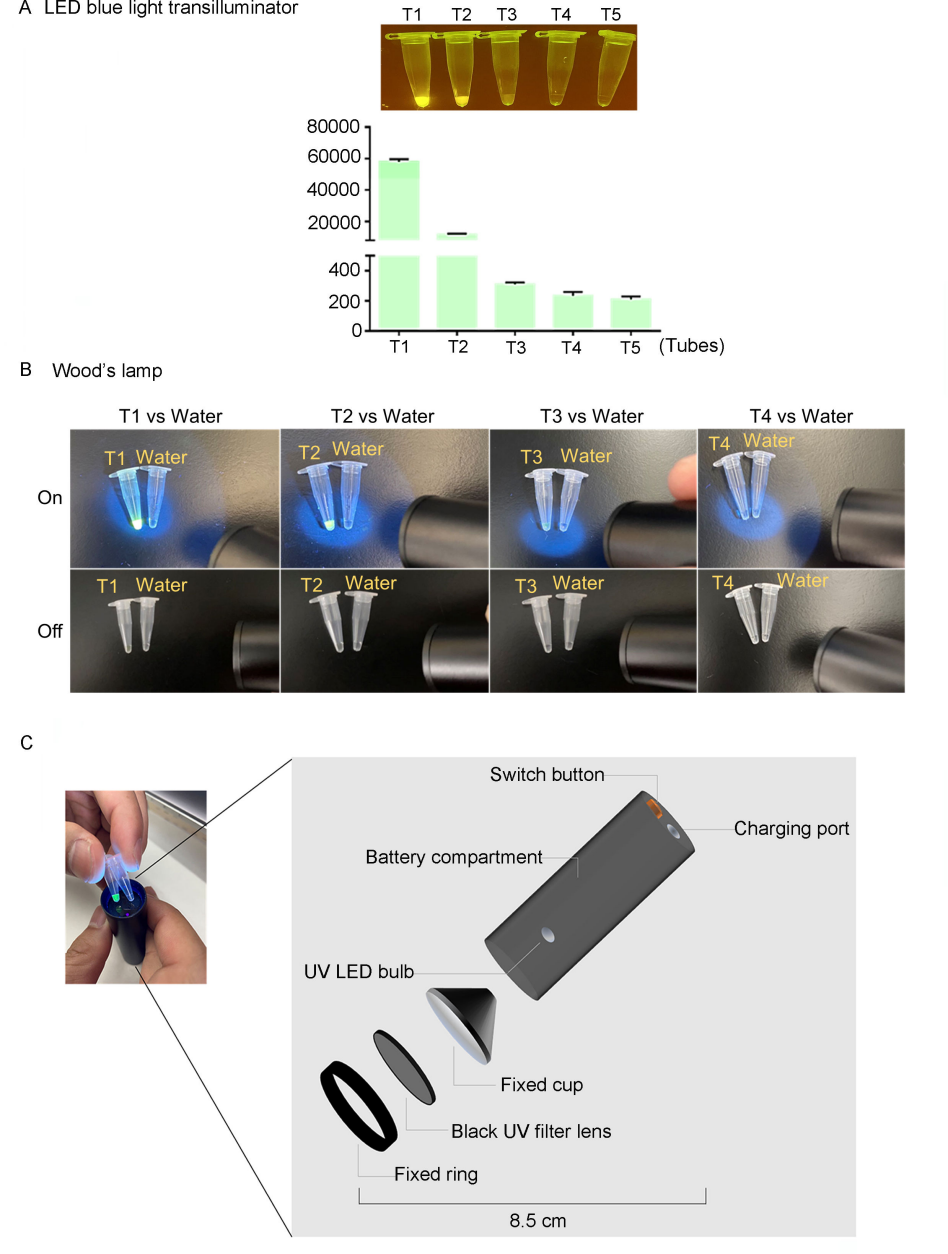
Performance comparison between LED blue light transilluminator and Wood’s lamp on visualizing fluorescence by the naked eye. (**A**) The fluorescence was fivefold serially diluted in five tubes (T1–T5), and the fluorescence intensity in each tube was measured and plotted. The detection range of the instrument is at 2,150–65,500. The representative images of tubes under an LED blue light transilluminator were present. (**B**) The tubes (T1–T4) were visualized by the naked eye using Wood’s lamp. The tube containing water was regarded as a control. A black background aids the visualization of by the naked eye. (**C**) The portable features of Wood’s lamp were displayed in detail.

The LED blue light transilluminator usually used in the lab relies on a power supply. To reduce the dependence on power and facilitate PRV detection, we compared the performance of the transilluminator and portable device Wood’s lamp on the tubes with diluted fluorescence. We fivefold serially diluted the 2 µM fluorescence with water in tested tubes until the fluorescence could not be visualized as a yellow-green color under a transilluminator or Wood’s lamp. We defined the visualization limit as the fluorescence intensity in the last tube in which the liquid is visible. We found that the fluorescence in Tube 1 (referred to as T1) and Tube 2 (T2) was very bright under both the transilluminator and Wood’s lamp. It is still slightly visible in Tube 3 (T3) but is invisible in Tube 4 (T4) and Tube 5 (T5) (Fig. 2A and B). The visualization limit based on the fluorescence intensity is around 3,099.66 ± 121.99 in T3 (Fig. 2A). The results suggested that the handy and battery-powered Wood’s lamp (Fig. 2C) is suitable for PRV detection.

### Optimization of the reaction conditions for MIRA-Cas12a assay

**Fig 3:**
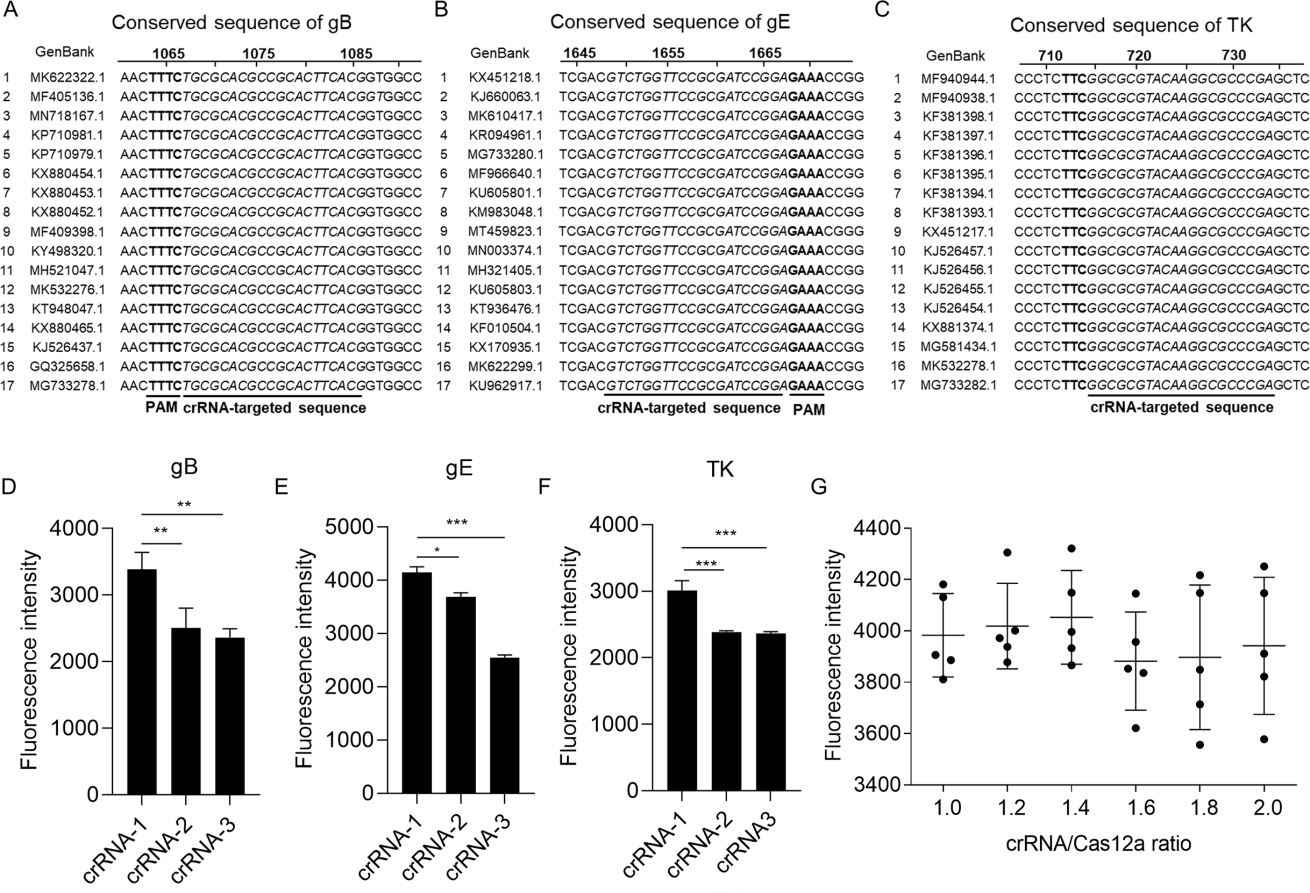
The crRNA selections and the optimization of the reaction conditions for MIRA-Cas12a assay. The conserved sequences of gB (**A**), gE (**B**), and TK (**C**) and the PAM and targeted sequence for crRNA1 are displayed. All sequences were obtained from the NCBI database and aligned by the MEGA program. To select a better crRNA, three crRNAs targeting gB (**D**), gE (**E**), or TK gene (**F**) were designed, and the fluorescence intensities were compared. The fluorescent signal produced by different crRNAs was collected with a qPCR instrument. Error bars represent the mean ± SD, and data from three experiments were included. *P < 0.05; **P < 0.01; and ***P < 0.001. (**G**) The fluorescence signals generated by different concentration ratios of LbCas12a to crRNA were compared to optimize the reaction conditions. The statistical analysis was performed using a one-way analysis of variance.

Seventeen genomes of PRV were downloaded from NCBI and aligned with each other by MEGA software. The most conserved sequences of gB (Fig. 3A), gE (Fig. 3B), and TK (Fig. 3C) were selected for crRNA design. The crRNA plays a central role in Cas12a-based detection (30 – 32). To screen out a highly efficient crRNA, we designed, synthesized, and purified three crRNAs for each gene and evaluated the efficiency of each crRNA by the intensity of fluorescence generated in Cas12a cleavage assay. Our results demonstrated that the gB-crRNA1 (Fig. 3D), gE-crRNA1 (Fig. 3E), and TK-crRNA1 (Fig. 3F) had better efficiency compared to the other two crRNAs. To further improve the efficiency of Cas12a cleavage, we compared the fluorescence intensities generated by the different ratios of crRNA to Cas12a. No statistical difference was observed between these crRNA/Cas12a ratios (1:1–2:1), and we selected the ratio of 1.4:1 with a relatively higher average intensity as the optimized ratio for the following detections (Fig. 3G).

**Fig 4:**
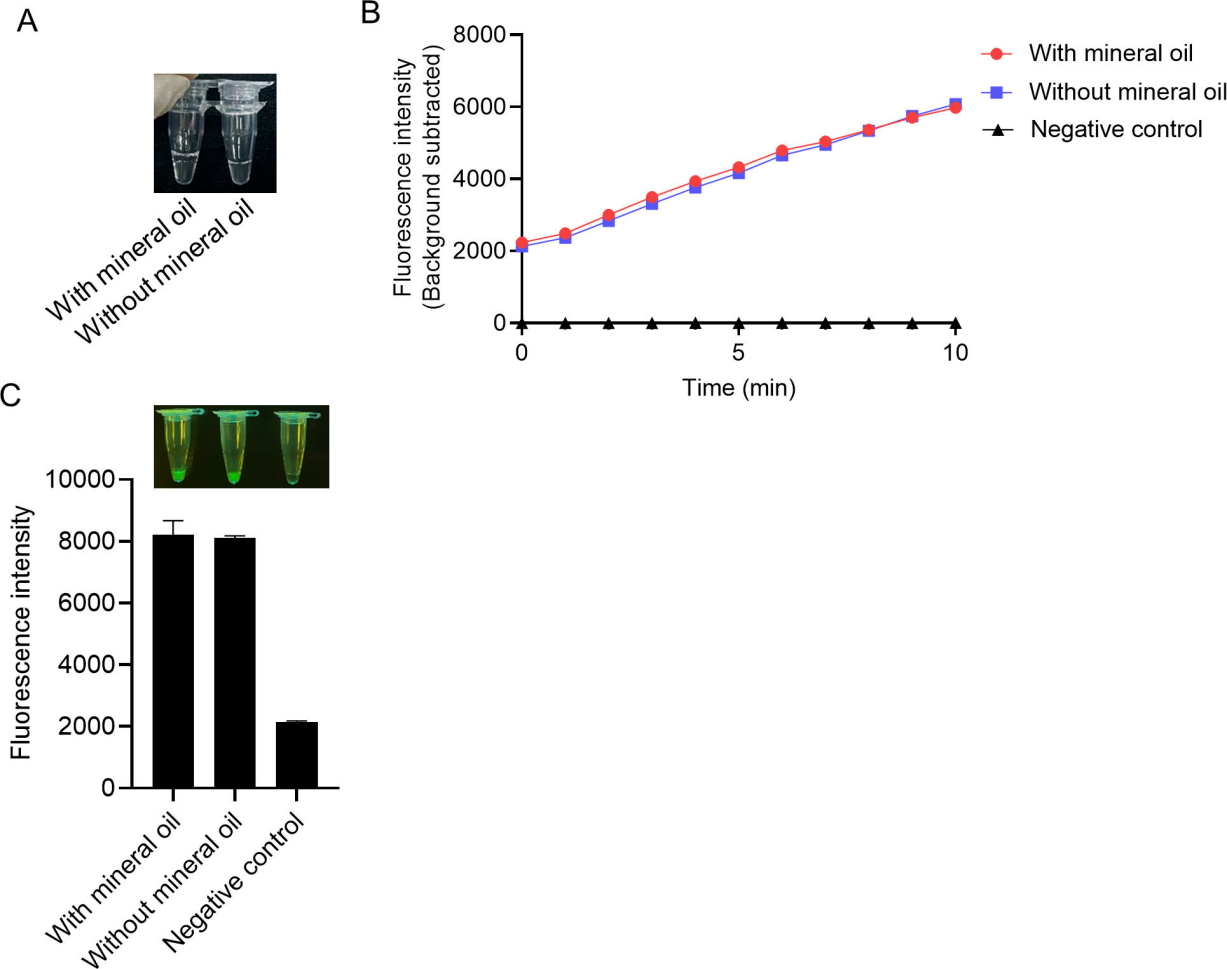
Adding mineral oil to prevent aerosol pollution did not affect MIRA-Cas12a detection. (**A**) Mineral oil was added to the MIRA reaction before amplification at 37°C. The reaction without mineral oil served as a control. (**B**) The fluorescence intensities during MIRA-Cas12a detection with or without mineral oil were monitored for 10 min. (**C**) After incubation for 10 min, the fluorescence intensities at the endpoint of the reaction with or without mineral oil were measured. The negative control indicates no target DNA added.

We used mineral oil in the viral nucleic acid detections to avoid the aerosol pollution that frequently happens in isothermal amplification (33 – 35). Firstly, we investigated whether mineral oil affects MIRA-Cas12a detection. We added 20-µL mineral oil into MIRA components (Fig. 4A) and monitored the fluorescence produced in the Cas12a cleavage. The real-time and the endpoint fluorescence intensity indicated no significant difference between the reactions with or without mineral oil (Fig. 4B and C). The results demonstrated that mineral oil did not affect the MIRA-Cas12a detection system.

### The sensitivity of MIRA-Cas12a assay on gB, gE, and TK of PRV

**Fig 5:**
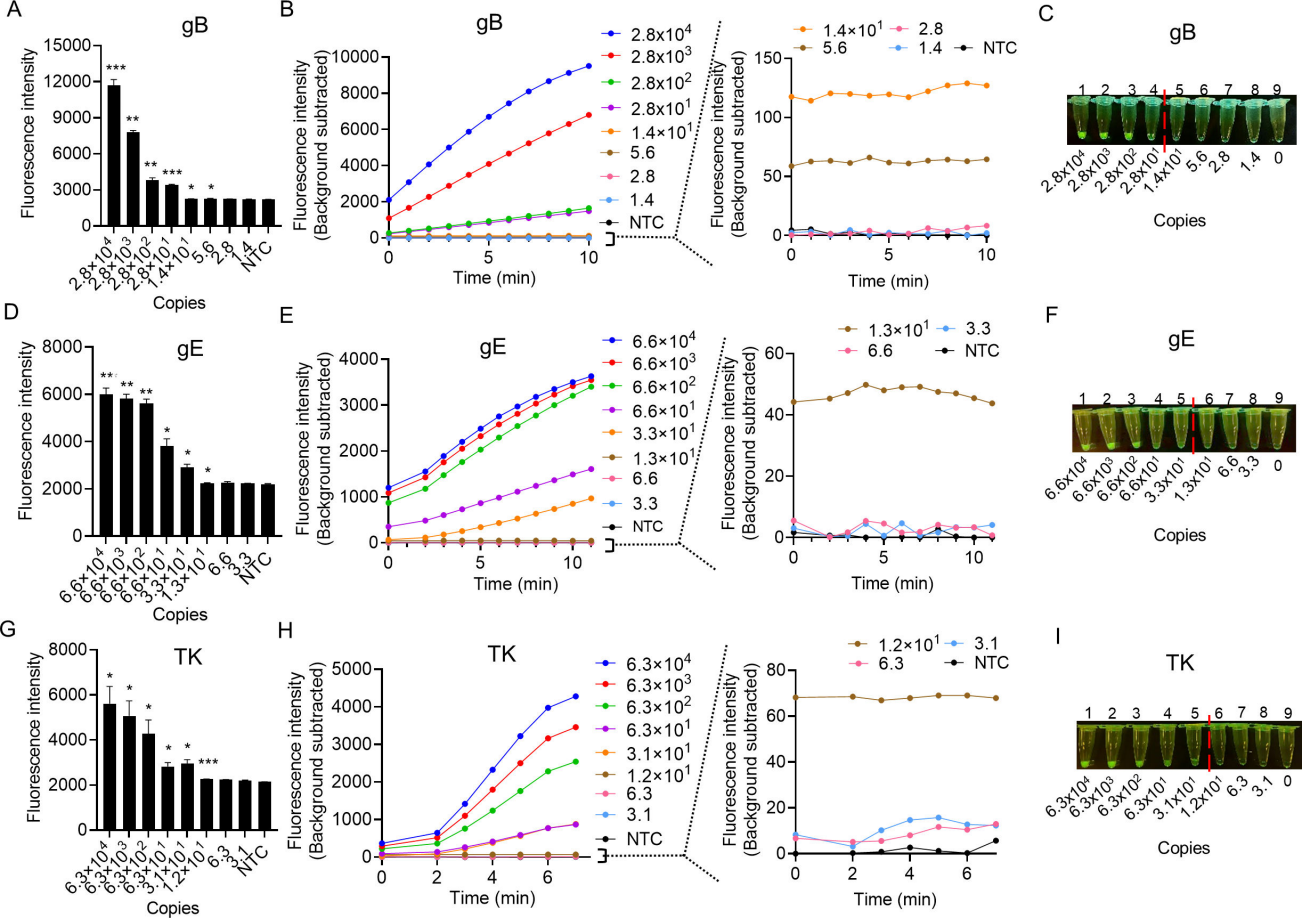
The sensitivity test of MIRA-Cas12a in detecting gB, gE, and TK genes. The plasmids containing target genes were serially diluted to determine the detection limit of the MIRA-Cas12a assay. The fluorescence intensities for gB (**A and B**), gE (**D and E**), and TK (**G and H**) detections were measured with a qPCR instrument. The fluorescence signal was observed under LED blue light with the naked eye for gB (**C**), gE (**F**), and TK (**I**) detections. The data represent the value of fluorescent signal obtained within 10 min and are presented as the mean ± SD (*n* ≥ 3). The statistical significance was generated by comparing the test samples with the NTC. The statistical analysis was performed using a two-tailed *t*-test. *P < 0.05; **P < 0.01; and ***P < 0.001. The red dotted lines indicate the boundary between positive and negative results identified by the naked eye under LED blue light. NTC, no template control.

**Fig 6:**
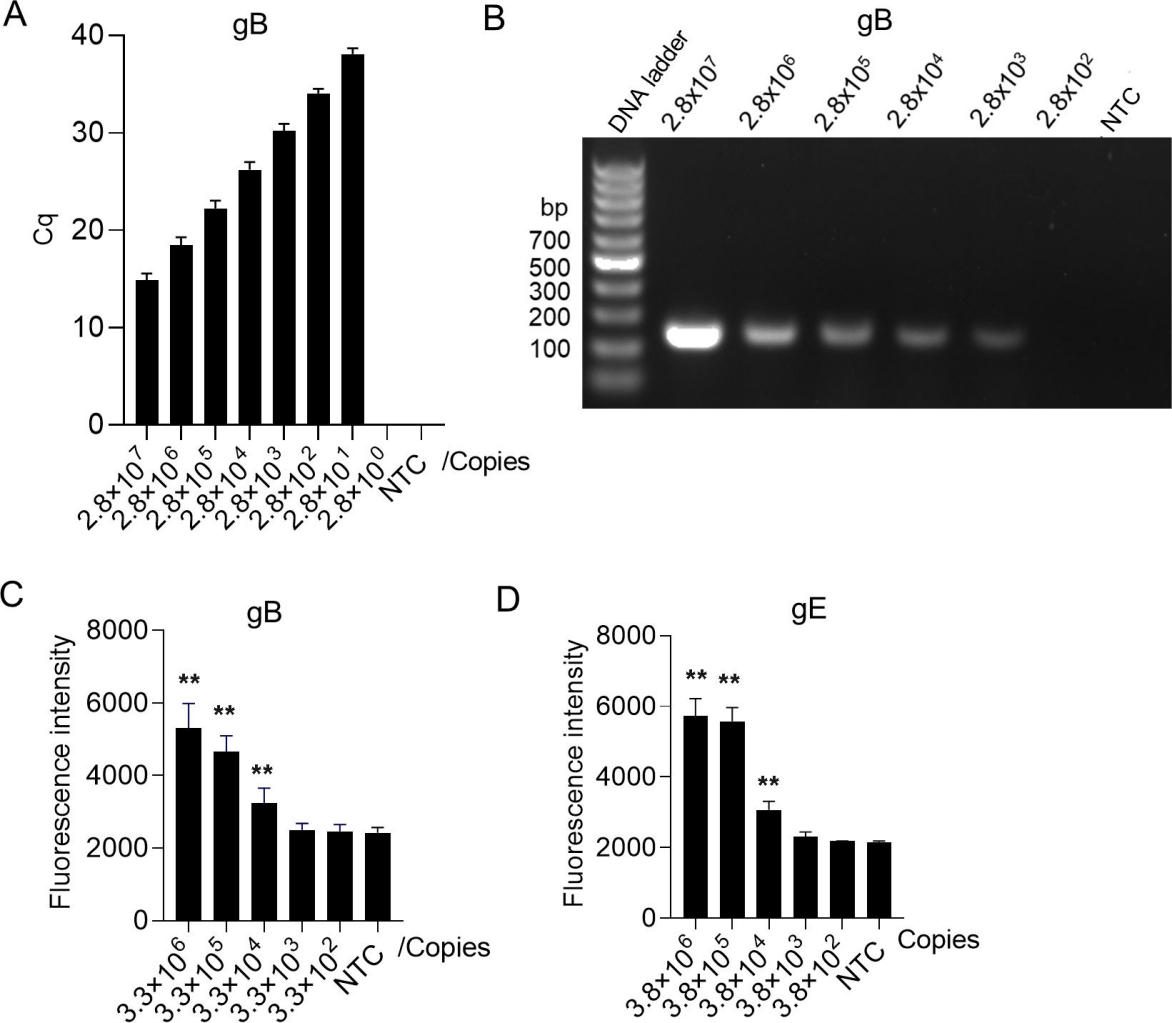
The sensitivity determination of qPCR, PCR, and sPAMC on PRV. The plasmid containing the gB gene was selected to determine the detection limit of qPCR (**A**) and PCR electrophoresis (**B**). The sPAMC assay for gB detection (**C**) and gE detection (**D**) used a suboptimal PAM (“CTTC”) and (“GTTG”), respectively. The fluorescence signals in the sPAMC assay were collected during incubation at 37°C for 15 min and calculated as mean  ±  SD (*n* = 5). The statistical significance was generated by comparing the test samples with the NTC. The statistical analysis was performed using a two-tailed *t*-test. *P < 0.05; **P < 0.01; and ***P < 0.001. NTC, no template control.

To determine the sensitivity of MIRA-Cas12a assay on gB, gE, and TK of PRV, the plasmids containing gB, gE, or TK were 10-fold serially diluted and used as targeted DNA. After incubating the plasmids with the MIRA reagents cocktail at 37°C for 15 min, the product was transferred to the preassembled Cas12a/crRNA complex. Once binding to the targeted DNA, the Cas12a/crRNA complex will cleave the surrounding ssDNA reporters and produce significant fluorescence signals. We first measured the real-time fluorescence with a qPCR instrument. The results showed that the MIRA-Cas12a system has a sensitivity of 5.6 copies/reaction on gB (Fig. 5A and B) if measured by qPCR instrument. The fluorescence can also be directly read out by the naked eyes under LED blue light after 10 min of incubation but with a sensitivity of 28 copies/reaction (Fig. 5C); the sensitivity on gE by qPCR instrument was 13 copies/reaction (Fig. 5D and E) and 33 copies/reaction by LED blue light (Fig. 5F); the sensitivity on TK by qPCR instrument was 12 copies/reaction (Fig. 5G and H) and 31 copies/reaction by LED blue light (Fig. 5I). We also compared the sensitivity of MIRA-Cas12a detection with the conventional detection methods qPCR and PCR. Consistent with previous studies (18 – 21), the detection limit for qPCR built in our research reached 28 copies/reaction, paralleled with the MIRA-Cas12a by the naked eyes (Fig. 6A). Moreover, qPCR and MIRA-Cas12a are much more sensitive than the PCR electrophoresis (Fig. 6B). Lu et al. found a sPAM (5′-VTTV-3′) for Cas12a and developed the sPAMC system. It allows isothermal amplification and Cas12a cleavage to react together in one tube, reducing the possibility of cross-contamination and showing a better efficiency than the canonical PAM (5′-TTTN-3′) (24). We used an sPAM (“CTTC”) and designed a crRNA targeting PRV’s gB and gE genes for one-pot detection. As shown in Fig. 6C and D, the detection limits for the sPAMC system were 3.3 × 10^4^ and 3.8 × 10^4^ copies/reaction for gB and gE, respectively. Our results demonstrated that the MIRA-Cas12a assay exhibited a comparable sensitivity with qPCR and was much more sensitive than PCR and sPAMC. What it needed are just a 37°C heater and a blue light source, rather than a bulky instrument like qPCR, which showed great potential in the detection of PRV. Additionally, we have detected cultured PRV diluents by qPCR and MIRA-Cas12a, and the result indicated that the sensitivity of MIRA-Cas12a is comparable to that of qPCR (Table 3).

**TABLE 3:** Comparison of qPCR and MIRA-Cas12a by detecting diluted PRV viruses cultured in the cells^*a*^

Methods	The titration of PRV (TCID_50_)							
	10^7^	10^6^	10^5^	10^4^	10^3^	10^2^	10^1^	NTC
qPCR (Cq)	15.6 ± 0.03	19.4 ± 0.11	23.8 ± 0.01	27.7 ± 0.16	30.9 ± 0.30	35.0	No value	No value
MIRA-Cas12a (fluorescence intensity)	50,808 ± 347***	44,693 ± 1,159***	20,325 ± 464***	9,039 ± 129***	4,770 ± 68***	2,693 ± 166**	2,612 ± 76*	2,313 ± 125

^*a*^ A two-tailed *t*-test was performed between dilutions and NTC. *P < 0.05; **P < 0.01; and ***P < 0.001. NTC, no template control.

### The performance of MIRA-Cas12a detection on the wild type, vaccines, and clinical samples of PRV

**Fig 7:**
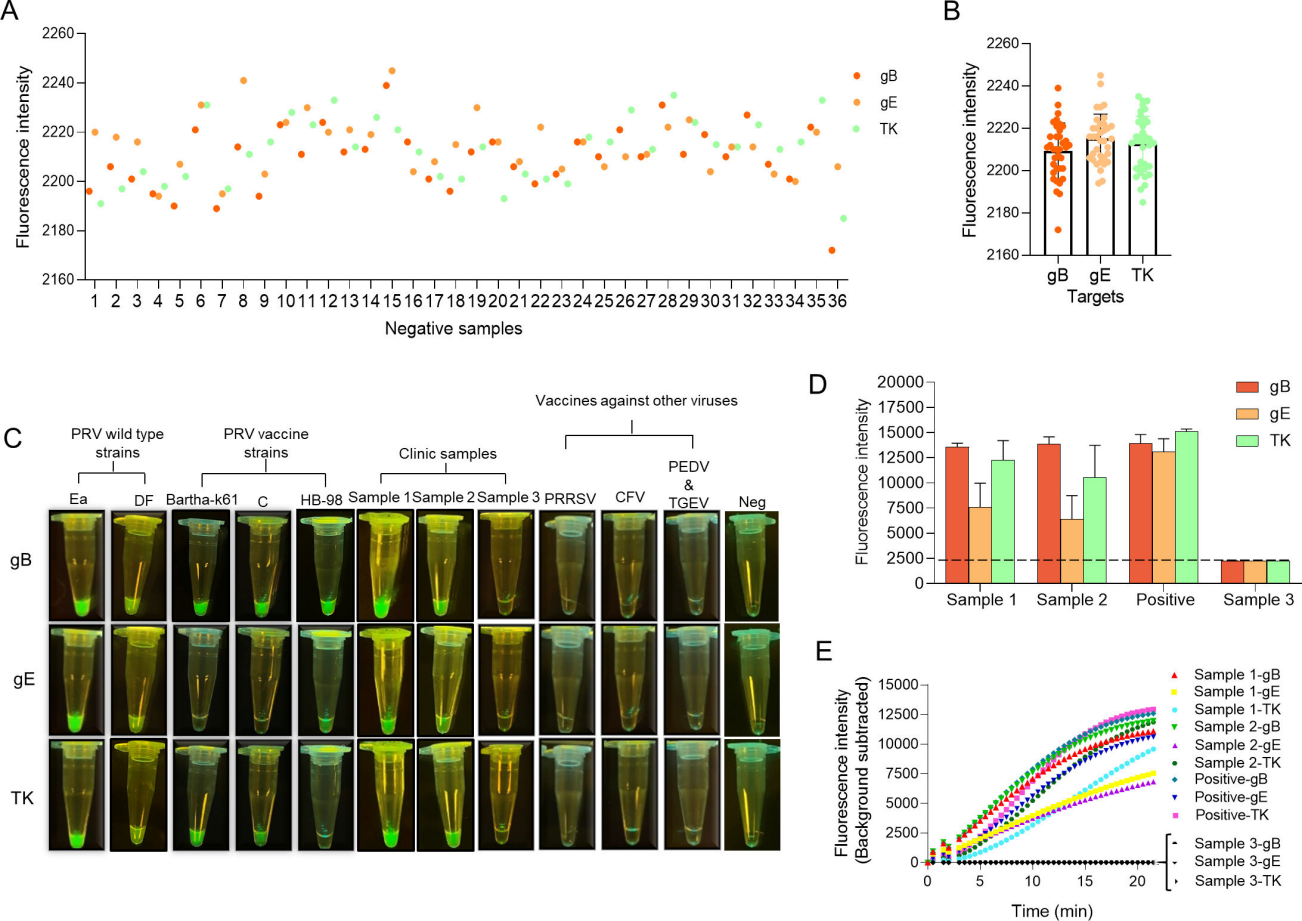
The differentiation of PRV vaccines and the wild-type strains by MIRA-Cas12a assay. (**A**) The MIRA-Cas12a assay detected the nucleic acid of negative clinical samples. The numbers 1–20 are nasopharyngeal swab samples collected from 20 swine; the numbers 21–36 are the samples from swine tissues, including liver (# 21–23), kidney (# 24–27), lung (# 28–30), intestine (# 31–33), and spleen (# 34–36). All the samples are from healthy swine. (**B**) The threshold between positive and negative results was determined by calculating the average fluorescence value produced in MIRA-Cas12a detection, which was expressed with mean ± SD (gB: 2,209.28 ± 13.24; gE: 2,215.08 ± 11.67; TK: 2,212.53 ± 13.11). (**C**) The wild-type PRV (Ea and DF strains), gE-deleted PRV vaccines (Bartha-k61 and C strain), TK-deleted vaccine (HB98 strain), and clinical samples (Samples 1–3) were used to test the performance of MIRA-Cas12a detection in the differential diagnosis. Samples 1 and 2 were from PRV-infected swine, and Sample 3 was from a PRV-uninfected swine, all determined by qPCR. Some common swine vaccines of PRRSV, CFV, and bivalent vaccine PEDV and TGEV were used to evaluate their specificity. Neg, reaction with no input. All the results were directly observed under LED blue light by the naked eyes. (**D**) The fluorescence generated in Samples 1 and 2 using MIRA-Cas12a assay were plotted. Positive control means that plasmids containing the target gene were added. The data are from three experiments and were represented as the mean ± SD. (**E**) The real-time curves of fluorescence signal in the MIRA-Cas12a detection on PRV clinical samples were plotted.

We have collected 36 clinical samples from healthy swine for MIRA-Cas12a detection to determine the threshold between negative and positive results. Based on the results shown in Fig. 7A and B, the thresholds of MIRA-Cas12a detection were 2,209.28 ± 13.24 for gB, 2,215.08 ± 11.67 for gE, and 2,212.53 ± 13.11 for TK. If the fluorescence intensity exceeds the value, it would be recognized as positive; otherwise, it would be negative. To further evaluate the performance of the MIRA-Cas12a detection, the wild-type PRV (Ea and DF strains), gE-deleted vaccine viruses (Bartha-k61 and C strains), TK-deleted vaccine viruses (HB98 strain), three clinical samples (Samples 1–3), and several vaccines against other swine viruses were used. We directly observed the generated fluorescence under LED blue light with the naked eye after the MIRA-Cas12a reaction. Tubes detecting gB showed strong fluorescence signal in the presence of wild-type PRV, the vaccine strains (Bartha-k61, C, and HB98), clinical Samples 1 and 2, but not in the presence of Sample 3, PRRSV vaccine, CFV vaccine, and the bivalent vaccine of PEDV and TGEV; tubes detecting gE presented unmistakable fluorescence signal in the presence of wild-type PRV, the vaccine strain HB98 and clinical Samples 1 and 2, but not in Sample 3, PRV vaccine Bartha-k61 strain and C strain, PRRSV vaccine, CFV vaccine, and the bivalent vaccine for PEDV and TGEV (Fig. 7C); tubes detecting TK displayed significant fluorescence signal in the presence of wild-type PRV, the PRV vaccine (Bartha-k61 strain and C strain) and clinical Samples 1 and 2, but not in sample 3, HB98, PRRSV vaccine, CFV vaccine, and the bivalent vaccine of PEDV and TGEV (Fig. 7C). These results indicated that the detection method was precise. Meanwhile, we monitored the fluorescence signals in Samples 1–3 and positive control during MIRA-Cas12a detection. The result showed that the intensities of fluorescence generated by incubation for 20 min to detect gB, gE, and TK were much higher in the tubes with clinical Sample 1 or Sample 2 than in tubes with negative control samples. (Fig. 7D). The fluorescence signal became stronger as the incubation time increased. Still, the real-time fluorescence intensity curves indicated that we could distinguish the positive samples as fast as in 10 min using a qPCR instrument (Fig. 7E). With less instrument, short time, and superior specificity, we demonstrated that MIRA-Cas12a detection was competent to detect and differentiate the vaccinated and wild-type PRV-infected swine.

## DISCUSSION

Many countries have enacted eradication policies to control the spread of pseudorabies, including immediate extinction of the PRV-infected, intensive regional vaccination and the DIVA program (3). In this study, we described a novel nucleic acid diagnostic based on MIRA-CRISPR-Cas12a for differential detection of the wild-type and vaccine of PRV. Usually, the live-attenuated PRV vaccine is developed by deleting gE or TK gene (Table 2). By targeting the conserved region within the gB, gE, and TK genes with optimized crRNA, the MIRA-Cas12a detection on gB, gE, and TK can identify most PRV vaccines. By parallelly testing several frequently used live-attenuated swine vaccines, including PRV vaccines (Bartha-k61 strain, C strain, and HB98 strain), PRRSV vaccine (CH-1R strain), CFV vaccine, and the bivalent vaccine for PEDV (AJ1102-R strain) and TGEV (WH-1R strain), we demonstrated that MIRA-Cas12a possessed a superior specificity of PRV detection, which potentiates its application in PRV detection. MIRA-Cas12a can be adaptable to apply in different scenarios. To determine whether PRV causes the illness of swine, we can detect the gB gene; to determine whether the swine has received PRV vaccines, we can detect TK or gE genes in one reaction; to decide which type of vaccine the swine has received, we can detect TK and gE genes, respectively. Identification of all three genes is advised if the related information is unclear or the condition is complicated.

MIRA-Cas12a is as sensitive as qPCR but takes only half of the time of qPCR. Compared to expensive and bulky instruments required for PCR or qPCR, MIRA-Cas12a detection only needs a 37°C incubator and an excitation source of UV blue light. Furthermore, the final results can be directly read out by the naked eye, while the qPCR result requires further analysis by skilled staff. The portable features and easy operations of MIRA-Cas12a detection allow it to be easily deployed for PRV diagnosis, even in resource-limited places. Using a novel type of PAM, the sPAMC system has achieved the target amplification and Cas12a cleavage simultaneously, which allows for detecting SARS-CoV-2 in 15 min with comparable sensitivity to qPCR (24). We compared the sensitivity of MIRA-Cas12a detection with the sPAMC and found that MIRA-Cas12a is more sensitive in PRV detection. The high GC content of the PRV genome sequence (~74%) (36, 37) is harmful to Cas12a/crRNA activity (38). The traditional PAM coupled with a complete target amplification like our two-step detection by MIRA-Cas12a might be more suitable for PRV detection than the sPAMC system, according to our results. Importantly, we inserted thymine in the forward primer to create a PAM when the conserved sequence lacked PAM for Cas12a recognition, which might eliminate the dependence of CRISPR-Cas-based detection systems on PAM (39 – 41), enabling us to designate any desirable site for CRISPR detection without the restriction of PAM. The strategy of inserting T coupled with MIRA here differs from the HOLMES system in which PAM was introduced by replacing the original bases of the targeted DNA with “TTT” using PCR (42). The different strategies in PAM introduction possibly lead to different DNA amplification efficiencies.

To facilitate the extinction of PRV, a reliable PRV detection should fulfill the following criteria: (i) it has comparable sensitivity with qPCR to capture the infected pigs; (ii) the procedure is fast enough to provide timely diagnostic information for the rule maker before the outbreak, especially for the highly contagious pathogens; and (iii) the procedure is simple enough to allow the test to be operated by the unskilled people in the frontline against PRV. In our study, by targeting the gB, gE, and TK genes of PRV, MIRA-Cas12a is able to differentiate the vaccinated and PRV-infected swine simply and rapidly with a sensitivity comparable to qPCR. Instead of the bulky instrument required for qPCR or the potential delay of antibody detection, MIRA-Cas12a provides a promising substitute for PRV detection, which would apply to pseudorabies prevention and eradication. Due to the unavailability of more clinically positive specimens, our study used only 36 clinically negative specimens and two positive ones. The viral titrations in the clinical samples vary a lot. It is worthy of testing the MIRA-Cas12a with more clinically positive specimens to evaluate the sensitivity and specificity more accurately.
